# Supplementary material for: Are carbon emissions trading and green financial instruments synergistic? -Comprehensive quantitative research based on content analysis
Source: PLoS One. 2024 Mar 7;19(3):e0298601. doi: 10.1371/journal.pone.0298601 (PMC10919601; doi:10.1371/journal.pone.0298601)
Supplement: S1 Data — (ZIP) [file pone.0298601.s001.zip › Data/地级市Z_F工作报告.zip!/╣π╢1⁄2/╔╪╣╪╩╨/└┤╫╘╣1⁄2╓┌║┼í╛┬φ┐╦╩2╛▌═°í┐_2013.docx]

各位代表：

我代表市人民政府向大会作政府工作报告，请予审议，并请政协各位委员和其他列席人员提出意见。

2012年工作回顾

2012年是国际金融危机爆发以来最困难的一年。市政府在市委的坚强领导下，在市人大及其常委会和市政协的监督、支持下，认真落实党中央、国务院和省委、省政府的决策部署，依靠全市人民，克服各种困难，应对复杂形势，开展“百项工程兴韶关”【1】活动，着力转方式、惠民生、增后劲，基本完成市十三届人大一次会议确定的2012年目标任务，经济社会平稳健康发展。

推进产业转型发展取得新成效。全年实现生产总值888.5亿元、增长9.8%，地方公共财政预算收入61.5亿元、增长14%，均高于全省平均增长水平。农业生产再创佳绩。新增标准农田2万亩，粮食产量实现“六连增”，仁化县被认定为国家现代农业示范区。工业保持较快增长。全年净增规模以上工业企业74户，年底达482户。服务业量质齐升。净增限额以上批发零售、住宿餐饮企业122家，年底达282家；南岭国家森林公园、珠玑古巷—梅关古道成为国家4A级景区；金融产业稳步发展，金融机构存贷款余额较快增长，汇丰银行、交通银行落户我市。投资力度加大。固定资产投资增长16.2%，发行企业债券14亿元，承接产业转移项目到位资金101.8亿元，新丰越堡水泥建成试产，乐昌城市广场投入使用，广乐高速公路建设加快推进，翁源华彩涂料城，以及曲江商务中心、南雄东方广场等商业综合体建设进展顺利，大广高速公路新丰段、韶能生物质能发电等项目开工建设，国电粤华煤矸石发电、南水水库供水工程、原曲仁矿棚户区改造等项目取得新进展。消费继续扩张。社会消费品零售总额增长14.1%。外贸逆势增长。进出口总额增长14.4%，增幅居全省第二位。创新驱动更加有力。专利申请量连续8年居全省山区市首位，高新技术产品产值增长18%，产学研工作荣获中国产学研结合促进奖。经济结构进一步优化。民营经济增加值增长13.6%，在经济总量中的比重首次超过50%。县域经济持续较快发展，增幅高于全市平均水平0.7个百分点。

统筹城乡发展取得新成绩。芙蓉新城建设扎实推进。芙蓉新城发展战略规划与控制性详细规划整合工作基本完成。赤水村安置区动工建设，芙蓉、车头、西联、下胡村安置区建设前期工作有序推进。碧桂园太阳城及五星级酒店、恒大地产、韶关联通、武警市支队作战指挥中心等项目加快推进。老城区得到优化提升。组织编制《韶关市主城区旧城改造及改造重点地区控制性详细规划整合》。通天塔重建竣工，百年东街基本建成，完成解放路和粤北医院门诊综合楼前道路的交通整治、林桥坑排水渠二期覆盖、韶关汽车客运南站主体工程等一批市政工程建设，北江大桥大修完成，二棉厂、油泵油嘴厂等一批“三旧”改造项目加快推进，市中心区通往曲江区客运班线实现公交化改造，投放新能源公交车38辆，完成1万多户“一户一表”自来水改造。新农村建设成效明显。全市硬化农村公路562公里，帮助低收入农户改造住房4870户，316个20户以下通电自然村实现广播电视“村村通”，首批2个名镇、20个名村、50个示范村创建工作初显成效。民生水利成效显著。乐昌峡水利枢纽开始发挥防洪效益，提前一年完成中央和省下达的农村饮水安全工程任务，累计解决72.8万农村人口饮水安全问题。扶贫开发“双到”工作圆满完成。全年落实帮扶资金4.44亿元、落实帮扶项目487个，贫困村集体生产性经营收入平均8.5万元，贫困户人均纯收入7546元。

生态文明建设取得新进展。乐昌、南雄、仁化、始兴、乳源五个县（市）被列入中央财政国家重点生态功能区均衡性转移支付范围。在全省率先完成136公里生态景观林带建设任务。完成造林38.8万亩，封山育林1162.4万亩。基本完成韶关国家森林公园生态休闲项目建设。启动了森林防火远程视频监控及预警系统一期建设。荣获省“林业生态市”称号。地质灾害防治和矿山资源整治加强，开展对重点工矿企业重金属污染综合整治和环境修复。产业转移工业园和中心镇污水处理设施建设全面推进。乡村清洁美工程深入开展，完成村庄整治363个，初步建立“户收、村集、镇运、县处置”的农村生活垃圾收运处置体系。全年淘汰190万吨落后水泥产能，减排二氧化硫约1万吨，规模以上工业增加值综合能耗下降16%，万元生产总值能耗下降4.2%。

改善民生和社会建设取得新进步。居民收入较快增长。全市农村居民人均纯收入、市区居民人均可支配收入分别增长15%和14%，企业退休人员人均养老金比上年增长10.1%。居民消费价格涨幅低于控制目标。民生投入持续增加。民生支出占财政支出的比重提高2.4个百分点，达69.6%。基本公共服务均等化水平提高。市政府承诺办好的8件实事和市政府各部门（单位）公开承诺为民办的49件实事基本完成。社保体系更加健全。实现城乡居民社会养老保险制度全覆盖，医疗保险城乡一体化及工伤、失业、生育保险和社会保障一卡通【2】建设扎实推进。开工建设保障性住房6362套，建成2829套。11万人次享受低保待遇，实施医疗救助8022人次。就业工作成绩显著。培训农村劳动力2.2万人，转移就业4.8万人。城镇新增就业5.4万人，城镇失业人员再就业4.2万人，城镇登记失业率2.75%。社会事业全面发展。3—5周岁幼儿学前三年毛入园率达92.9%，义务教育规范化学校覆盖率达93.6%，户籍人口高中阶段毛入学率达92%以上，武江区、曲江区、始兴县、乳源瑶族自治县成为广东省教育强县（区）。新型农村合作医疗参合率100%，基本公共卫生服务均等化各项年度任务全面完成，政府办基层医疗卫生机构全面实施基本药物制度，公立医院改革试点工作稳妥推进，医疗服务能力得到提升。首次开展农村实用技术人才职称评审工作。首次接受全国文明城市创建“两测”国检【3】成绩排名靠前，完成国家和省文化惠民工程任务。建成29个乡镇农民健身广场。成功创建第九届全国双拥模范城。全国人口计生综合改革示范市工作和省市共建韶关食品药品安全示范区工作取得新进展。妇女儿童、老龄、民族、宗教、对台、外事、侨务、港澳、司法、史志、统计、审计、物价、档案、供销、新闻、出版、气象、水文、人防、防震减灾、广播电视、应急管理、无线电管理与信息化等工作取得新进步。深入开展“三打两建”，启动平安韶关创建，社会管理得到加强。安全生产形势总体平稳。

政府自身建设取得新成果。扎实转变政府职能。深化行政审批制度改革和县（市、区）党政机构改革迈出重要步伐，出台第一批行政审批事项改革目录，取消行政审批事项113项、转移31项、下放61项、委托2项。推进要素市场改革，组建了公共资源交易中心和监管机构。加强廉政风险防控。全面落实领导干部廉政责任制和“五个一”制度，成立市预防腐败局。推进网络问政和政务公开，加强纠风工作，解决了一批关系群众切身利益的问题。强化效能监察。完善市直机关绩效考评、突出贡献奖、县域科学发展观考核等制度。同时，自觉接受监督，支持人大代表和政协委员履行职责。实施人大议案1件、政协建议案3件。办理代表建议77件和委员提案112件，办复率和办结率均为100%，代表、委员普遍表示满意或基本满意。

各位代表，上述成绩来之不易，这是全市人民团结奋斗的结果。在此，我代表市政府，向全市人民，向驻韶人民解放军和武警官兵，向各民主党派、人民团体、社会各界人士表示崇高的敬意！向关心、支持韶关发展的港澳台同胞和海内外朋友表示衷心的感谢！

在看到成绩的同时，我们也清醒地看到面临的困难和问题： 2012年我市经济增长和实际利用外资没有实现预期目标；经济发展方式仍然比较粗放，资源、能源、环境约束加剧，产业结构有待进一步优化；工业发展后劲不强，第三产业发展偏慢，农业现代化水平不高；中心城市带动力不强，城乡差距依然较大；社会建设任务繁重，民生质量有待进一步提高。对此，我们一定要增强忧患意识，采取有力措施加以解决。

2013年工作安排

今年政府工作的总体要求是：坚持发展这个硬道理，以科学发展观和习近平总书记视察我省提出的“三个定位、两个率先”【4】目标为指引，认真落实党的十八大精神和省委、省政府决策部署，切实增强加快发展的紧迫感，坚定信心，抓住机遇，加快转变经济发展方式，以新型工业化和新型城镇化双轮驱动，积极推进信息化、农业现代化和基本公共服务均等化，着力深化改革开放，着力实施“双转移”战略，着力保障和改善民生，实现经济增长质量和人民幸福指数同步提升。

综合考虑国家和我省经济发展趋势向好，以及国内外经济发展环境复杂、不确定性增加等多种有利和不利因素，今年全市经济社会发展主要预期目标是：生产总值增长11%以上；人均生产总值增长10%；地方公共财政预算收入增长11%以上；固定资产投资增长16%；社会消费品零售总额增长15%；城镇居民人均可支配收入增长9%，农村居民人均纯收入增长10%；居民消费价格指数涨幅控制在4%左右；城镇登记失业率控制在3%以内；单位生产总值能耗下降3.9%，完成省下达的节能减排等各项任务。

围绕上述目标，我们将更加注重解放思想、深化改革，用党的十八大和习近平总书记视察广东重要讲话精神武装头脑，着力破除妨碍科学发展的思想束缚，以更大的勇气和智慧推进符合韶关实际的改革，着力建设大市场、好社会、强政府，在改革中获取发展的最大红利；我们将更加注重转变发展方式、提高发展质量，突出以人为本、绿色转型、共建共享，追求利税效果更好、创新能力更强、环境代价更小、资源消耗更少、群众受益更广的更为协调、可持续的包容性增长，用发展的办法解决前进中的矛盾和问题；我们将更加注重联系群众、转变作风，深入基层、摸索规律、解决问题，务实进取、不尚空谈、廉洁自律，以政府作风的转变促进各项工作的进步。

发展不足是我市当前最大的问题，加快发展是解决我市所有问题的关键。今年突出抓好五个方面的工作：

一、加强经济建设

在推动韶关科学发展的全局里，必须扭住经济建设这个中心不动摇，在加速经济发展的过程中，坚持质量为先，充分体现富民惠民取向。

改善对经济运行的调控，保持经济较快增长的势头。从我市作为欠发达地区的实际出发，在投资、消费、出口三大经济增长驱动力中：首先要突出投资拉动的关键作用。加大质量招商力度。整合招商资源，创新专业招商工作机制，努力“招大商、招好商”。加强与珠三角的有机联系，强化产业对接，加大承接产业转移力度。大力推进乡贤反哺工程【5】，吸引外出乡贤回韶投资兴业。加大项目工作力度。开展“项目建设年”活动。加强项目前期工作和项目储备，建成和不断完善市重点项目储备库。实施更加积极的财政政策，集中财力上一批事关我市长远发展的基础设施、公共服务、民生保障关键项目，并相应加强政策引导和服务，撬动和带动信贷资金、社会资本加大投入。突出抓社会资本的投资到位，积极协调解决项目用地、规划、环评等前期工作，促成签约项目尽快落地动工，在建项目尽快建成投产、达产、达效。其次要发挥扩大消费需求的重要作用。优化消费环境，培育消费热点，繁荣活跃城乡市场。合理布局建设区域商务中心，积极发展社区商业服务网点。加快培育健康、文化、养老服务等消费热点。积极推广信贷、租赁等新型消费模式。推进“农超对接”【6】，加强平价商店建设管理。继续实施“万村千乡”市场工程和“新网工程”，拓展农家店综合服务功能，大力开拓农村市场。通过完善公积金贷款政策等措施，引导释放社会住房需求，拉动衍生消费需求。组织专业商品展会，满足群众需求。此外，还要加大外贸工作力度。加强口岸基础设施建设，优化通关环境，做优“铁海联运”，培育出口品牌，努力开拓国际市场，培育外贸新增长点。

加快转型升级，构建现代产业体系。积极实施《广东省韶关市资源枯竭城市转型发展规划》。坚持绿色发展。培育发展绿色健康产业，积极发展健康卫生、智慧生活、绿色环保、休闲旅游等幸福导向型产业，加快把生态优势转化为发展优势，积极探索依靠生态加快发展的路子。坚持集约发展。从我市既要加快发展又要调整结构的“双重任务”出发，在激活存量的同时，更多地在增量上做文章，以增量提升促存量优化，加快产业规模化、集约化、集群化进程。一是大力发展现代农业，加强三农工作。提高农业综合生产能力和城乡防灾减灾能力。认真落实中央一号文件精神。落实强农惠农政策，建设高标准基本农田，完善农田水利设施，保障主要农产品有效供给。促进政策性涉农保险业务发展。推进气象现代化。提高农业产业化水平。积极组织农民，大力发展农业龙头企业、专业合作社、种养大户和家庭农场，新增市级以上农业龙头企业10家、农民专业合作社100家。完善基层农业技术推广体系，培养现代新型农民。加快土地流转，促进土地适度规模经营；大力推进绿色种植，发展特色精品农业；提高农产品精深加工水平。改进包装，加强配送，搞活农产品流通。提高农业发展载体水平。抓好现代农业园区建设，打造现代农业示范基地。强化林业建设。大力发展乡土珍贵阔叶树、油茶、竹子、绿化苗木等林业主导产业，打造450万亩特色产业基地，有效调控竹木采伐加工规模，采取措施大力发展林下经济。大力创建幸福村居【7】。统筹谋划全市农村自然村建、转、撤、并，支持两不具备村庄整村搬迁；鼓励引导社会资本参与村庄建设。新建400公里通自然村路面硬化公路。开展全省“公交一卡通”【8】在农村客运领域的试点应用。优化扶贫方式。启动新一轮扶贫开发，支持民族地区、革命老区、贫困地区、水库移民区加快发展。二是加大“工业强市”力度。促进工业集群发展。从实际出发，明确我市各地工业发展的产业定位。促进企业按产业分工协作关系集聚发展，着力打造、补足、延伸、强化产业链，推进机械装备制造、钢铁、有色金属、烟草、电力、新型建材、精细化工、稀土、生物医药、食品饮料、纺织服装、家具和玩具等产业健康发展，重点发展带动力强的整机装备、成套设备、智能装备和终端产品生产企业。加大工业项目推进力度。加快韶钢优特钢、翁源华彩涂料城、韶关发电厂、韶能生物质能发电、东阳光生物制药、氟化工等在建项目建设，争取华电（南雄）热电联产、国电粤华煤矸石发电、南水水库供水工程、曲江台泥等项目动工建设。调整工业结构，优化工业存量。继续推动韶钢、韶关烟厂、东阳光、丹冶、凡口矿、大宝山矿等现有优势企业技改创新和韶冶环保搬迁，同时积极扶持韶关液压、韶铸、中机重工、韶瑞重工、正星车轮、东南轴承、北江纺织等一批潜力较大的企业做大做强。总结青岛啤酒入主我市活力啤酒公司和世界矿山机械巨头芬兰美卓集团兼并韶瑞重工的经验，引导工业企业与国内外行业领先企业合作或重组。引导鼓励扶持个体户发展为企业、规模以下工业企业发展为规模以上工业企业。制定优惠政策，支持有条件的工业企业加快上市。落实支持中小微企业【9】发展的政策措施，培育中小企业创新产业化示范基地，缓解中小企业融资难题，促进中小企业加快发展。优化工业园区载体建设。重点推进省级产业转移工业园以及莞韶华南钢铁科技产业园、稀土产业园规划建设。加快扩园工作，扩大园区自主权，加大园区基础设施和配套设施投入，加强公共服务平台建设，扩展园区服务功能，将园区建成更具竞争力、吸引力的工业新城。大力支持各县（市、区）工业园区建设，培育一批优质县域工业园。三是加快服务业发展。加大旅游资源开发力度，促进旅游业率先跨越发展。积极引入有诚意、有实力、有经验的旅游开发战略投资者，加大南雄奥威斯、新丰云髻山等旅游重点项目推进力度，提高旅游产业化水平。重点建设环丹霞山旅游产业园，整合资源、改革体制，培育做大丹霞山景区市场主体。丹霞山巴寨景区免费开放100天【10】。积极推进农村旅游社区建设，发展农家乐旅游。引导旅游业与其它产业融合发展，发展节庆、商务会议等旅游新业态。举办好啤酒节等节庆旅游活动，进一步打响徒步穿越丹霞山品牌。整合本地吃、住、行、游、购、娱等要素，打造有竞争力的旅游产品，并与周边市合作推广精品旅游线路，着力提高游客过夜停留比重。加快完善市区通往各主要景区景点的高等级快速旅游交通网络。积极发展社区服务业。重视发挥金融业的作用。加强政府、企业与金融机构的交流，引入更多金融机构，支持金融机构扩大和创新业务，引导商业银行提高贷存比。完善农村金融服务体系。大力发展商贸流通业。推进专业市场、商业综合体、星级和商务酒店等商贸项目建设，发展带动力强的连锁经营龙头企业。争取物流业发展有新突破。建设物流园区，发展现代物流配送和专业物流中心。培育供应链一体化管理现代化物流龙头企业，推进成品油、钢材、香烟、煤炭、矿石、农副产品等大宗商品物流项目建设，推动物流业与工商业联动发展。积极发展电子商务。加快发展网络零售、行业电子商务、综合性电子商务平台，壮大网商队伍。

推进城镇化进程，着力城市扩容提质。以韶关市区和各县（市）城区为重点加快城市建设，引导农村人口向城镇、周边人口向市区聚集，推进城镇化和农民市民化，加快建设粤北区域中心城市，增强辐射带动周边发展的能力。完善创新流动人口管理服务，完善异地务工人员积分制入户城镇、参与社会管理、享受公共服务等制度。要把芙蓉新城建设作为城镇化的重中之重来抓。抓好芙蓉新城扩区域和组建管委会工作，按照打造机械装备企业总部基地、生态旅游集散地、区域行政文化中心、宜居宜业家园的定位合理安排开发时序。坚持农民优先，完成赤水村安置房建设，开工其他4个村安置房项目；坚持道路优先，全面推进芙蓉大道、芙蓉隧道、南华路、32号路、百旺路、滨江路等新城骨干路网建设，完善新城道路基本框架；坚持公建优先，加快学校、医院、文化场馆和便民服务中心、韶关联通、移动服务大楼及武警市支队作战指挥中心等建设；坚持产业骨干项目优先，加快金融街、中央商务区、总部基地、专业市场、城市综合体【11】等产业带动项目的建设。继续优化提升老城区。改造老城区东西南北四大出口，推进上饶路、新兴路、群康路、东环线、移山路等道路建设，提高市区交通畅达水平。大力推进“三旧”改造。加大违法违章建筑整治力度。大力推进城市管理科学化、精细化、信息化。加强交通等基础设施建设。加快广乐、大广高速公路、赣韶铁路和武广高铁乐昌东站建设。推进仁深、汕昆高速公路和北江航道“五改三”前期工作。韶关汽车客运南站投入使用。建设智能化公交综合管理系统，优化加密连接新老城区的公交线路。推进CNG气站【12】建设。建设西气东输天然气市区门站。加速创新型城市建设。强化创新平台，改善创新环境，推进技术创新成果产业化。加强各县（市）城区和有条件的中心镇规划建设。强化规划协调和衔接，全面开展市域城镇体系规划和县域村镇体系规划，优化功能定位和产业布局，大力培育发展中心镇和特色专业镇。积极促进城市要素和公共资源向农村延伸。

二、加强文化建设

深化文化体制改革，大力发展公共文化服务，推动文化事业和文化产业发展，充分发挥文化引领风尚、教育人民、服务社会、推动发展的作用。

加强社会主义核心价值观教育。着力推进以社会公德、职业道德、家庭美德、个人品德、诚实守信为主要内容的公民道德建设工程，加强和改进未成年人思想道德建设，大力倡导“厚于德、诚于信、敏于行”的新时期广东精神。加快公共文体服务体系建设。推进文化骨干工程和公共文化基础设施建设，加快建设芙蓉新城图书馆、文化馆、档案馆。完成文化信息资源共享、农村电影放映、广播电视“户户通”、县镇数字影院建设等工程。促进文化产业发展。加快培育广播电视、新闻出版、演艺娱乐、平面传媒、创意设计、信息网络等产业。推进大南华文化旅游创意产业园、金鸡岭文化产业园等建设，以重点文化项目建设带动文化产业发展。加强文化市场监管。规范文化市场和网络文化管理，深入开展“扫黄打非”。推进正版软件在公务部门的应用。深化文化体制改革。加大市直文艺院团改革力度。推进文明城市建设。推进全国文明城市、国家历史文化名城创建和历史文化名镇、名村的保护。做好国家卫生城市复审通过工作。建立市、县两级非物质文化遗产保护名录和数据库。深入开展群众性文体活动。努力打造韶关群众文体活动品牌。办好世界张氏总会第六届恳亲大会和纪念“六祖”惠能示寂1300周年系列活动。实施全民健身计划。建成21个乡镇农民体育健身工程，推进机关、企事业单位体育设施向社会开放。推进市运会和市中小学运动会合二为一。力争在省第三届体育大会上取得好成绩。

三、加强社会建设

以保障和改善民生为重点，充分发挥政府在推动社会管理创新中的主导作用，推进和谐韶关建设。

着力保障和改善民生。合理调整收入分配关系，强化工资支付保障机制，维护劳动者合法权益。实施积极的就业创业政策，抓好农村劳动力技能培训转移就业工作，推动实现更高质量的就业。加强养老保险、医疗保险、低保、社会救助、优抚安置、住房保障和残疾人康复等工作，完善城乡统筹的社会保障体系。启动第十届全国双拥模范城创建。加大解决农村集体经济发展用地历史遗留问题工作力度。坚持教育优先发展，促进教育公平，努力创建省教育强市。完成学前教育三年行动计划（2011—2013年），进一步推进学前教育健康发展。推动异地务工人员子女平等接受教育。推进城乡义务教育均衡发展，加强农村寄宿制学校建设，着力解决“小校园大班额”现象；促进学校内涵发展，加强校长和教师队伍建设。推进高等教育整合资源，提高办学质量和水平。加大改革力度，推动技工教育、中职教育从数量扩张向质量提升转变，推进校园对接产业园工程，推行招工即入学的“校企双制”【13】联合办学。完成市特殊教育学校搬迁改造。提升医疗卫生等公共服务水平。健全农村三级医疗卫生服务网络和城市社区卫生服务体系，抓好乳源瑶族自治县公立医院改革试点。巩固城乡基层医疗卫生机构综合改革成果，推进“平价医院、平价诊室、平价药包”建设。合理配置卫生资源，推进粤北区域医疗服务中心建设，推动中医药强市战略实施；开展城乡环境卫生整治，加强城乡居民健康教育工作。大力发展慈善事业。关注民生价格，保持价格总水平基本稳定。

着力加强和创新社会管理。全面推进创建平安韶关工作，加大社会信用体系和市场监管体系建设力度。制定扶持社会组织发展的政策措施，建设社会组织培育基地，加快培育发展社会组织，推进社会组织去行政化、去垄断化改革。创新社会利益和矛盾调处机制，建立社会建设信息网络平台，及早化解社会矛盾和冲突。完善立体化社会治安防控体系，提升应急管理水平，增强群众安全感和满意度。全面开展城乡社区建设，完善社区管理和服务体制，打造专业化社区工作者队伍，推进社区服务信息化标准化。继续推进食品药品安全示范区创建，以及人口计生、安全生产、民族团结进步事业等工作。建立社会建设工作综合考核评价体系，优化社会事业发展方式，推动教育、医疗等公共服务资源优化整合与布局调整，提高公共服务质量。创新社会建设投融资机制，在加大财政对社会建设投入的同时，积极吸引民间资源进入社会建设领域。

今年，要在继续做好市政府以往承诺民生工作的同时，办好10件关系民生的实事：1、推行森林保险；2、开展自然灾害公众责任保险；3、建立扶持中小企业融资机制；4、实施广乐高速公路北连接线犁市互通黄岗至十里亭路段改（扩）建工程；5、实施韶南大道百旺大桥到南华寺路段改（扩）建工程；6、实施工业西路内涝整治工程；7、建设韶关国家森林公园8公里健身步道；8、建设北江中学新运动场和综合大楼；9、建设五里亭桥底和北江源群众文体活动广场；10、为全市敬老院安装太阳能热水器。

四、加强生态建设

大力实施绿色发展战略和主体功能区规划，正确处理发展与保护的关系，面上加强保护、点上有序开发，打造“蓝天、青山、碧水、洁净、美丽”的宜居宜业环境，推进绿色低碳、可持续发展，促进生态效益和经济效益“双赢”。

着力巩固生态屏障。大力推进封山育林，抓好生态景观林带、森林碳汇【14】、森林进城围城等重点林业生态工程建设和绿道网建设，加快石漠化、水土流失、地质灾害和荒废矿区综合治理，强化对河沙采集的管理，加强自然保护区、生态公益林、湿地、江河水库保护。

着力强化环境保护。认真落实环保规划，抓好重点区域、重点行业和重要领域的规划环评。加大减排工作力度，重点抓好污水处理项目、城市污水管网建设以及机动车和规模化畜禽养殖场减排措施的落实。完善落后产能退出机制，完成小水泥等落后产能淘汰任务。加强重金属污染防治。深入开展农村环境综合整治和生态示范村（镇）创建，建设美丽乡村。

节约集约利用资源。实施供电与产出挂钩政策，加强对高耗能行业和企业的调控，实施差别水价政策，积极推广LED照明产品【15】。制定全市土地利用水平年度考核办法，强化“以亩产论英雄”的理念，推行企业用地与产出挂钩，依法征收土地税费，探索土地出让合同附加项目质量和建设进度条件，促进节约集约用地。加强土地批后、供后监管，严格耕地保护。优化国土空间开发，做好市重大发展平台和重点项目用地规划调整工作，保障重点项目建设用地。抓好城乡建设用地增减挂钩【16】试点工作。争取土地利用改革先行先试，充分利用低丘缓坡和废弃工矿地保障发展。大力发展循环经济，加强再生资源回收利用管理。

五、加强政府建设

我们将牢固树立“政务就是服务”的理念，重点在依法行政、深化改革、改进作风、廉政建设、人才支撑上再造政务服务新优势。

严格依法行政。自觉接受市人大及其常委会的法律监督和市政协的民主监督，办好人大议案、代表建议和政协提案。虚心听取各民主党派、工商联、无党派人士和各人民团体的意见建议。完善市政府工作规则和会议制度，制定《韶关市重大行政决策程序规定》，把公众参与、专家论证、风险评估、合法性审查和集体讨论决定纳入决策程序。建立重大行政决策咨询论证专家库。深入推行行政执法责任制，完善行政执法程序，进一步减少和规范行政执法自由裁量权。推进行政执法与刑事司法衔接，规范执法行为。公开政务信息，完善网络问政。尽力解决历史遗留问题，公平对待市场主体，打造诚信法制政府。

深化改革。以转变政府职能为核心，继续深化行政审批制度改革，清理、调整和压缩市级行政审批事项，减少、下放和向社会组织转移行政审批职能。实施审批目录管理，落实首问负责、限时办结、服务承诺、并联审批等制度，优化政务服务流程，缩短审批时限。建成网上服务大厅，加强市县镇三级行政社会服务体系建设，提高服务效能。优化创新政府提供公共服务机制，有序推进政府向社会组织购买公共服务【17】工作，降低行政成本，提高服务质量。深化企业投资管理体制改革和商事登记制度改革。推进山区县农村综合改革、富县强镇和简政强镇事权改革。完成县（市、区）党政机构改革。大力推进事业单位分类改革，开展法人治理结构试点。加强政府投资管理体制改革。加大市属国企改革力度，支持其与行业领先企业合作或重组，完善法人治理结构和经营机制。落实协税护税机制，加强税收征管。

改进工作作风。领导干部要带头转变作风，坚持密切联系群众。市政府领导和政府所属单位一把手要率先深入基层，针对影响我市科学发展和群众利益的突出问题扎实调研，按照市委、市政府谋划确定的发展大思路，结合本职工作实际进行具体谋划，按程序形成具体工作目标和措施，通过现场办公等形式及时协调决策，狠抓督促落实。简化、优化政府部门检查评比，更加重视工作结果，并在相对集中时段进行，减轻基层负担。大力推进电子政务建设，全面实施OA办公系统【18】办公，以信息技术提高政务透明度，倒逼政府工作人员提高工作效率。转变文风会风，践行开短会、讲短话、发短文，严控会议和表彰庆典等活动。整顿机关作风，坚决整治庸懒散奢，严查和制止行政不作为、乱作为、慢作为行为。

加强廉政建设。进一步落实党风廉政建设责任制和一把手述责述廉、“五个一”等制度，深入开展廉政风险防控和行政权力公开透明运行机制建设，构建惩防体系。全面加快公共资源交易平台建设，健全工作规则和流程，探索公共工程和政府网上采购招标、资格后审机制。加强政府工程监督，对经批准的项目严格投资评审，按照批复概算组织实施。优化财政支出，严控行政成本，发文规范税费减免。加强审计监督，除对政府部门的审计外，还要扩大对事业单位、重点国企等单位的审计，创新开展对政府工程、政府采购和公共资产处置招标的审计，清查纠正限制公平竞争、损害公众利益的行为。加强政府内部廉政监察、执法监察、效能监察、纠风和督办工作，强化重大工程、重点行业、重点领域和重要环节的监管。

强化人才支撑。必须把人才与事业紧密结合起来，完善聘用、开发、激励约束等激活人才资源的体制机制，让合适的人干合适的事，让合适的人干好合适的事。积极实施符合韶关发展阶段性要求的人才工程，着力储备、培养、吸引、用好人才，重点引进和培养较高层次适用人才，加快科技创新团队和产业创新联盟建设。制定文件完善政府公务员和事业单位人员录用的基本条件和办法，提高选录工作公平性。

各位代表，让我们在省委、省政府和市委的正确领导下，解放思想、实事求是、群策群力、攻坚克难，为建设幸福美好韶关而努力奋斗！
